# Ketogenic Diet: A New Light Shining on Old but Gold Biochemistry

**DOI:** 10.3390/nu11102497

**Published:** 2019-10-17

**Authors:** Raffaella Longo, Carolina Peri, Dalma Cricrì, Lara Coppi, Donatella Caruso, Nico Mitro, Emma De Fabiani, Maurizio Crestani

**Affiliations:** Dipartimento di Scienze Farmacologiche e Biomolecolari, Università degli Studi di Milano, via Balzaretti 9, 20133 Milano, Italia, , , , , , emma.defabiani@unimi.it (E.D.F.)

**Keywords:** ketogenic diet, ketone body regulation, epigenetics, epilepsy, cancer, neurological disorders, metabolic disorders

## Abstract

Diets low in carbohydrates and proteins and enriched in fat stimulate the hepatic synthesis of ketone bodies (KB). These molecules are used as alternative fuel for energy production in target tissues. The synthesis and utilization of KB are tightly regulated both at transcriptional and hormonal levels. The nuclear receptor peroxisome proliferator activated receptor α (PPARα), currently recognized as one of the master regulators of ketogenesis, integrates nutritional signals to the activation of transcriptional networks regulating fatty acid β-oxidation and ketogenesis. New factors, such as circadian rhythms and paracrine signals, are emerging as important aspects of this metabolic regulation. However, KB are currently considered not only as energy substrates but also as signaling molecules. β-hydroxybutyrate has been identified as class I histone deacetylase inhibitor, thus establishing a connection between products of hepatic lipid metabolism and epigenetics. Ketogenic diets (KD) are currently used to treat different forms of infantile epilepsy, also caused by genetic defects such as Glut1 and Pyruvate Dehydrogenase Deficiency Syndromes. However, several researchers are now focusing on the possibility to use KD in other diseases, such as cancer, neurological and metabolic disorders. Nonetheless, clear-cut evidence of the efficacy of KD in other disorders remains to be provided in order to suggest the adoption of such diets to metabolic-related pathologies.

## 1. Introduction

The ketogenic diet (KD) is a dietary regimen intended to increase ketone bodies (KB) synthesis and utilization. To push metabolism towards ketogenesis, KD is enriched in fat and very poor in carbohydrates and adequate in protein; thereby the classic dietary pyramid of macronutrients composition is completely overturned with respect to normal dietary recommendations. This aspect deeply impacts compliance and quality of life of subjects treated with KD. Ketogenesis, mostly occurring in the liver, leads to the synthesis of acetoacetate (ACA) and β-hydroxybutyrate (βOHB), two main KB, from mitochondrial acetyl-CoA pool. This pathway is usually active during fasting or prolonged exercise, when hepatic gluconeogenesis uses oxaloacetate from alanine, lactate and tricarboxylic acid (TCA) cycle to produce glucose. Therefore, acetyl-CoA from β-oxidation exceeds the level of oxaloacetate and is not further condensed to citrate, thus becoming precursor for KB. Traditionally, ketogenesis has been seen simply as a “spill-over” pathway that distributes KB as energy molecules to other tissues during fasting or prolonged exercise. However, ketogenesis also regenerates mitochondrial NADH to NAD^+^ via βOHB dehydrogenase [1]. The key, limiting step of ketogenesis is catalyzed by hydroxy-methyl-glutaryl-CoA synthase 2 (HMGCS2). Utilization of ketone bodies from non-hepatic tissues occurs in several tissues through ketolysis, and the rate-limiting enzyme is 3-oxoacid-transferase 1 (OXCT1), also known as also known as succinyl-CoA transferase (SCOT) or thiophorase. These pathways are finely regulated at transcriptional and hormonal level. Interestingly, KB are not simply energy substrates but also act as signaling molecules. βOHB has been recognized as an epigenetic regulator, by acting as class I histone deacetylase (HDAC) inhibitor. KD is currently used to treat epilepsy, particularly infantile refractory forms and it is the standard of care for glucose transporter 1 deficiency syndrome (GLUT1 DS) and pyruvate dehydrogenase deficiency syndrome (PDH DS). The exact mechanism of action of KD is heterogeneous, spanning from inhibition of glycolysis and of the conversion of its product to lipid metabolism, to regulation of mitochondrial metabolism. Moreover, KB can also regulate neuronal activity and transmission through different mechanisms. As KB act at many different levels, other possible therapeutic uses of KD are under consideration, such as in neurological disorders, cancer and metabolic diseases. In this review we will describe how ketogenesis is tightly regulated with a focus on novel aspects of regulation. We will also discuss possible mechanisms of action of KD and finally, we will review current efforts to use KD in several diseases.

## 2. Overview of the Biochemical Reactions of KB Synthesis and Utilization

Ketogenesis, starting either from fatty acid oxidation or oxidation of ketogenic amino acids, leads to the formation of ketone bodies, three distinct molecules known as βOHB, (ACA) and acetone. They represent circulating energy molecules during fasting or prolonged exercise. Most of the biosynthetic process occurs in the mitochondria of hepatocytes, even though a small production may be found in other tissues, like kidney epithelia, astrocytes and enterocytes [2]. In the initial phase of fasting, tissues rely primarily on glucose metabolism and glycogen stored in muscle and liver is depleted first. After a prolonged fast, fatty acids are mobilized from adipose tissue to the liver and they undergo β-oxidation to produce acetyl-CoA that enters the TCA cycle. Under normal conditions, acetyl-CoA from fatty acid β-oxidation is further oxidized via the TCA and, then, the reduced coenzymes NADH and FADH_2_ allow the production of energy by the electron transport chain in the mitochondria coupled to ATP synthesis (oxidative phosphorylation, OXPHOS). However, TCA cycle cannot handle the large amount of acetyl-CoA derived from fatty acid β-oxidation due to a combination of factors: low levels of insulin increase fatty acids release and, together, enzymes required for ketone body synthesis and utilization. Simultaneously, diversion of oxaloacetate to feed gluconeogenesis in the liver leads to low activity of TCA cycle due to reduced amounts of intermediates. This increases acetyl-CoA levels, which can be used as a substrate for ketone body synthesis [3]. (Nelson, D. Cox M. Lehninger, Principles of Biochemistry).

### 2.1. Ketone Body Production

Acetyl-CoA derived from fatty acid β-oxidation is the substrate for the first step of ketogenesis: acetoacetyl-CoA thiolase (ACAT1) catalyses the condensation of two molecules of acetyl-CoA to form acetoacetyl-CoA. Mitochondrial hydroxymethyl glutaryl-CoA synthase (HMGCS2), the rate-limiting enzyme of the pathway, promotes the addition of a third acetyl-CoA molecule to form 3-hydroxy-3-methylglutaryl-CoA (HMG-CoA). The first ketone body, acetoacetate, is then produced by HMG-CoA lyase (HMGCL). ACA is the common precursor of the other two ketone bodies: it is mostly reduced to βOHB by NADH-dependent β-hydroxybutyrate dehydrogenase (BDH). The third ketone body derives from the spontaneous decarboxylation of ACA in a volatile product, acetone, which is largely excreted through the lungs. The mitochondrial compartmentalization of ketogenic enzymes allows acetyl-CoA (necessary for ketogenesis) to separate from the acetyl-CoA addressed to anabolic processes (de novo fatty acids and cholesterol synthesis)

### 2.2. Ketone Body Utilization (Ketolysis)

**Figure 1:**
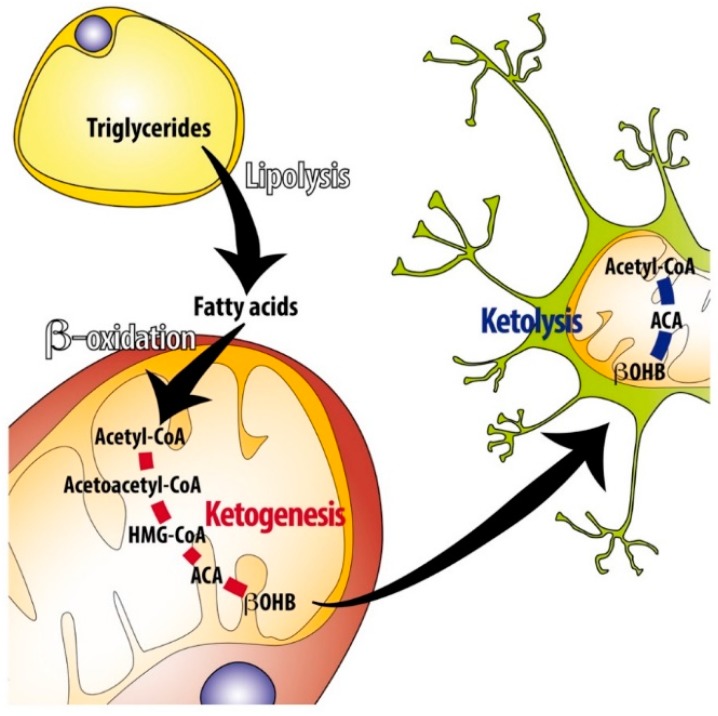
Overview of ketogenesis and ketolysis. During fasting or prolonged exercise fatty acids are released from adipose tissue via lipolysis of triglycerides. Fatty acids can be internalized and oxidized in hepatocytes. Subsequently, acetyl-CoA derived from fatty acid β-oxidation is used as precursor for ketone body synthesis (acetoacetate [ACA] and β-hydroxybutyrate [βOHB]). βOHB is released in the circulation and, after internalization in extra-hepatic tissues (in this figure brain is represented by the neuron) via MCT1 (monocarboxylate transporter 1) is used as a substrate to obtain acetyl-CoA and to produce energy via TCA (tricarboxylic acid cycle) cycle and electron transport chain.

Circulating ketone bodies are taken up by target tissues and used to regain energy via oxidation. Ketolysis occurs in the mitochondria of the many extrahepatic organs that absorb ketone bodies from the blood through MCT1, the monocarboxylate transporter 1, which also plays a role in KB release from the liver. Then BDH1, the same enzyme occurring in the last step of ketogenesis, converts βOHB back to acetoacetate. Acetoacetate is then conjugated with Coenzyme A to form acetoacetyl-CoA. The reaction is catalyzed by OXCT1, also known as SCOT or thiophorase, which transfers CoA from succinyl-CoA to acetoacetate. This is the rate-determining step in ketolysis. The liver does not express OXCT1, which prevents a futile cycle of KB synthesis and degradation. The third and final step of ketolysis is the generation of two molecules of acetyl-CoA from acetoacetyl-CoA by the reversible enzyme ACAT1 (Acetyl-CoA Acetyltransferase 1). The acetyl-CoA formed is then oxidized in non-hepatic tissues via the TCA cycle and the respiratory chain for ATP synthesis [4] (Figure 1).

## 3. Regulation of Ketogenesis

The production of ketone bodies relies on the amount of free fatty acids available for hepatic fatty acid β-oxydation (FAO). Therefore, lipolysis in adipocytes is the first control point. This pathway is activated by β-adrenergic stimulation during fasting, whereas insulin inhibits this pathway [5]. Another physiologically important regulatory mechanism is the modulation of flux of acyl-CoA through carnitine acyltransferase 1 (CPT1), which is activated when malonyl-CoA levels decrease. Malonyl-CoA is the product of the reaction catalyzed by two isoforms of acetyl-CoA carboxylase (ACC1 and ACC2). CPT1 activity is crucial to supply fatty acids to mitochondrial FAO, which leads to acetyl-CoA for KB production [5]. The third control site in ketogenesis is the mitochondrial 3-hydroxy-3-methylglutaryl CoA synthase (HMG-CoA synthase), which catalyzes the irreversible first step of ketone body synthesis [6]. Quantitative analysis of liver mitochondrial phosphoproteome revealed HMCGS2 as one of the most highly phosphorylated protein, with five out of ten phosphorylation sites exhibiting changes related to mouse strain, age, or obesity/diabetic status [7]. In particular, Ser456 phosphorylation by protein kinase A (PKA) and/or casein kinase 2 (CK2), increases HMGCS2 activity. Authors also treated HEK293 cells transfected with *HMGCS2* construct with ketogenic medium (depleted of FBS and supplemented with sodium octanoate and PPARα agonist Wy14,643). This treatment increased p-Ser456 and enzyme activity, suggesting that this post-translational modification may mediate enhanced KB synthesis upon ketogenic demand. Each step can be influenced by hormonal regulation (like change in the molar ratio of glucagon: insulin) and also by transcriptional factors.

### 3.1. Hormonal Regulation

Insulin, glucagon, cortisol, catecholamines and growth hormone are the principal hormones involved in the endocrine regulation of ketogenesis [2].

**Figure 2:**
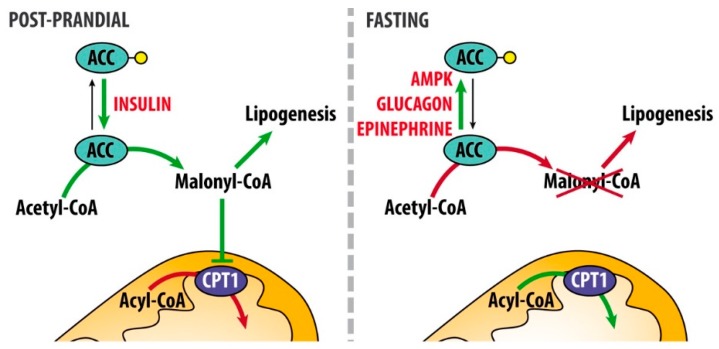
Hormonal regulation of malonyl-CoA synthesis. After a meal (left panel), insulin release promotes the dephosphorylation (phosphate group is here represented by the yellow dot) and activation of acetyl-CoA carboxylase, ACC, therefore increasing malonyl-CoA levels. This metabolite is the precursor for fatty acid synthesis but it also inhibits carnitin-palmitoyl transferase 1 (CPT1) activity and, consequently, fatty acid β-oxidation. Under this condition ketogenesis does not occur because of the lack of production of mitochondrial acetyl-CoA from fatty acid β-oxidation. During fasting (right panel), AMP kinase (AMPK), glucagon and epinephrine contribute to increase the phosphorylation and inactivation of ACC activity. Therefore, CPT1 activity is unlocked and fatty acid β-oxidation occurs, thus producing high amount of mitochondrial acetyl-CoA, which can be used as precursor for ketone body production. Green arrows indicate active reactions under a given condition; red arrows represent inactive reactions.

Post-prandially, insulin lowers ketone body concentration by complementary mechanisms. First, it inhibits lipolysis by suppressing the activity of hormone-sensitive lipase (HSL) thus reducing the release of fatty acids from adipocytes and their availability for ketogenesis. Second, it differentially regulates lipogenesis and fatty acid β-oxidation: it stimulates dephosphorylation and activation of the rate-limiting step of de novo fatty acid synthesis ACC, resulting in elevated malonyl-CoA levels. Elevated malonyl-CoA levels reduce fatty acid β-oxidation via inhibition of CPT1 and, consequently, the translocation of acyl-CoA to mitochondrial matrix, leading to reduced ketone body formation in liver and other ketogenic tissues (Figure 2). Of note, ACC has two main isoforms: ACC1 (*Acaca*) is mainly involved in fatty acid synthesis, ACC2 (*Acacb*) is engaged in regulation of fatty acid β-oxidation. Therefore, regulation of ACC enzyme activity by insulin both promotes de novo lipogenesis and blocks fatty acid β-oxidation (from Berg JM, Tymoczko JL, Stryer L. Biochemistry. 5th edition. New York: W H Freeman; 2002. Section 22.5, Acetyl Coenzyme A Carboxylase Plays a Key Role in Controlling Fatty Acid Metabolism. Available at: https://www.ncbi.nlm.nih.gov/books/NBK22381). Third, insulin promotes glucose uptake and oxidation. On the other hand, the reduction of circulating insulin levels is the principal triggering event for accelerating ketogenesis. When blood glucose levels are low, glucagon leads to the reduction of malonyl-CoA levels, by contributing to the phosphorylation and inactivation of acetyl-CoA carboxylase 2 (*Acacb*). As a consequence, malonyl-CoA-mediated inhibition of CPT1 is removed, long-chain acyl-CoA enter the mitochondrial matrix and undergo FAO and thus favoring ketogenesis (Figure 2). Along with glucagon, cortisol, and catecholamines, growth hormones also stimulate lipolysis, releasing free fatty acids. AMP kinase (AMPK), which is activated when energy charge is low (i.e., increased levels of 5′-AMP), is the enzyme directly phosphorylating ACC1 and ACC2, leading to their inactivation.

### 3.2. Transcriptional Regulation

Adaptation to prolonged periods of energy deprivation requires a metabolic rewiring that is regulated also at the transcriptional level. Peroxisome proliferator receptor α (PPARα) is a transcription factor included in the steroid/thyroid/retinoid receptor superfamily and it is a nuclear lipid-activated receptor thus functioning as lipid sensor specifically controlling fatty acid catabolism [8]. PPARα activates transcription by binding to DNA sequence elements, known as peroxisome proliferator response elements (PPREs), as a heterodimer with the retinoid X receptor (RXR), the nuclear receptor for 9-*cis*-retinoic acid. The promoters of PPAR responsive genes contain PPRE. When PPARα-RXR complex is activated by PPARα ligands and/or 9-*cis*-retinoic acid, the retinoid and fatty acid signaling pathways contribute in modulating lipid metabolism [9]. Fatty acids and their derivatives are PPARα endogenous ligands. This nuclear receptor activates the transcription of genes involved in lipid metabolism and ketone body biosynthesis and import [10]. Examples of PPARα target genes are: fatty acid binding protein 4 (*Fabp4*), carnitine palmitoyltransferase 1A (*Cpt1a*), peroxisomal acyl-CoA oxidase (*Acox)*, mitochondrial long chain acyl-CoA dehydrogenases (*Acadl*) and medium chain acyl-CoA dehydrogenases (*Acadm*), and also the ketogenic enzyme, *Hmgcs2* [2]. Studies in PPARα null mice corroborate its important role in regulating the response to prolonged food deprivation. After 24 h fasting, these animals showed hypoglycemia, low levels of circulating ketone bodies, hypothermia and high levels of free fatty acids in plasma, suggesting that fatty acid uptake and oxidation are inhibited. These observations underline the importance of PPARα to stimulate hepatic FAO and ketogenesis [11]. It has been recently reported that upon fasting extra-hepatic mast cells release histamine in portal circulation [12]. By activating H_1_ receptors, histamine induces the production of one of the most potent natural PPARα agonist, oleolylethanolamide, which enhances ketogenesis, thus demonstrating that integration of hormonal and transcriptional regulations finely tunes ketogenesis. PPARα transcriptional rewiring upon KD exposure occurs in tissue-specific manner. KD feeding increased levels of free fatty acids and expression of PPARα target genes in the liver in a non-circadian fashion [13]. In contrast, in the gut, KD induced rhythmic accumulation of PPARα in the nucleus, which paralleled mouse food intake and increased PPARα binding to rhythmic genes, accompanied by increased accumulation of free fatty acids during nocturnal period. Authors also demonstrated that KD influenced chromatin recruitment of core clock transcription factor BMAL1 (brain and muscle ARNT-like 1) in hepatic clock-controlled genes but not in the intestinal ileal cells. In contrast, KD induced ileal rhythmic transcriptional reprogramming that was dependent upon oscillation of PPARα nuclear accumulation. Strikingly, cyclic nuclear accumulation of PPARα and PPARα-driven gene expression were mirrored by oscillation of βOHB serum levels.

Another component of core clock, PER2 (period circadian clock 2), may regulate ketogenesis. In time-restricted feeding PER2 regulates *Cpt1a* and, via indirect mechanism, *Hmgcs2* expression, increasing the levels of βOHB, which, in turn, activates the so-called food anticipation, a series of manifestations anticipating feeding time [14]. Of note, βOHB supplementation was not sufficient to restore food anticipation in liver *Per2^-/-^* mice, indicating this KB as one of the components regulating the systemic response that helps preparing to food intake.

### 3.3. FGF21 is an Endocrine Regulator of the Ketotic State

Recent evidence has shown that PPARα plays a broader role during starvation and KD by inducing fibroblast growth factor 21 (FGF21) in the liver. PPARα directly stimulates the expression of FGF21, which acts as an autocrine/endocrine factor and contributes to the expression of genes relevant for ketogenesis. FGF21, a member of the FGF superfamily, is an endocrine hormone, expressed mostly in the liver and is a mediator of the pleiotropic actions of PPARα. Experiments with gain-of-function and loss-of-function approaches highlighted that FGF21 regulates the metabolic adaptation to starvation, like lipolysis and ketogenesis and, moreover, it is required for maximal hepatic lipid oxidation and ketogenesis induced by KD [15,16]. In the loss-of-function study with adenoviral knockdown of hepatic *Fgf21*, authors reported reduction of fatty acid oxidation and serum KB levels, increased lipemia, and lipid accumulation in the liver [16]. This trend results from the altered expression of key genes governing lipid and ketone metabolism. Gain-of-function experiments showed that FGF21 regulates different processes associated with fasting, like lipolysis in white adipose tissue and ketogenesis [15]. These two complementary approaches demonstrate that the PPARα-FGF21 axis plays an important role in the response to prolonged period of food deprivation or ketogenic diet by regulating ketogenesis.

### 3.4. The Ketogenic Factor FGF21 as Pharmacological Target

Since FGF21 regulates important aspect of metabolism, it represents an attractive potential pharmaceutical target for treatment of obesity, type 2 diabetes, and dyslipidemia. Studies on the pharmacological potential of FGF21 reported that, in obese rodents, it causes weight loss and improves insulin sensitivity, it reverses hepatic steatosis, and lowers circulating and hepatic triglycerides and cholesterol [17]. Similar results were obtained also in obese insulin-resistant primate models [18]. However, the translation of these observations to humans is still ongoing. In two recent clinical trials, long-acting FGF21 derivatives lowered body weight and insulin levels in obese subjects with type 2 diabetes [19]. These results indicate that FGF21 might represent a promising clinical candidate and that FGF21-based therapies may give efficient results for the treatment of metabolic disorders. However, longer studies are needed to assess beneficial as well as possible unwanted effects of FGF21 in humans.

### 3.5. Epigenetic Regulation by Ketone Bodies

KB have long been considered a source of energy in the bloodstream, however more recently, their role as signaling molecules has been discovered. β-hydroxybutyrate is also an endogenous inhibitor of class I histone deacetylases (HDACs) [4]. HDACs are epigenome modifiers that, by removing acetyl groups from histone tails, compact chromatin. Generally, hyperacetylation is associated with increased transcriptional activity, whereas hypoacetylation is associated to repression of gene expression. HDACs have been grouped in four classes based on their homology to yeast HDACs. Class I HDACs (HDACs 1–3 and 8) are broadly expressed and localized especially in the nucleus [20]. Class II HDACs (HDACs 4–7, 9 and 10) can be found both in the cytoplasm or in the nucleus, depending on their phosphorylation status, and show minimal histone deacetylase activity [21]. Class III HDACs are also known as sirtuins: they are NAD^+^-dependent deacetylases, which cleave NAD^+^ to create an ADP-ribose acceptor for the acetyl group, and hence their activity is sensitive to changes in the intracellular NAD^+^/NADH ratio. Mammalian sirtuins have been connected to a wide range of processes and they also play a central role in glucose and lipid metabolism [22]. Little is known about the function of HDAC11, the single member of class IV HDAC in mammals [23].

βOHB specifically inhibit HDAC1, 3 and 4 in vitro with an IC_50_ of 2–5 mM. Cells treated with βOHB showed histone hyperacetylation directly dependent to HDAC inhibition [24]. In the same study, mice infused with βOHB showed histone hyperacetylation (particularly H3K9ac and H3K14ac) in the kidney [24]. Histone acetylation induced by βOHB was associated with the induction of *Foxo3a* and *Mt2*. The forkhead box O3 (*Foxo3a*) is a transcription factor leading to cell quiescence and antioxidant capacity [25] and Metallothionein (*Mt2*) also protects against oxidative stress [26]. The authors demonstrated that βOHB induced histone hyperacetylation at the promoters of antioxidant genes by inhibiting HDAC1 and 2 [24]. The circadian response to KD indicates oscillation of serum βOHB accompanied by cyclic inhibition of HDAC activity only in the gut and, consequently, increased H3K9/K14 acetylation at PPREs of *Hmgcs2* promoter [13]. These data demonstrate that epigenetic regulation by βOHB deeply impacts metabolic circadian response to KD in a tissue-specific fashion. Interestingly, βOHB levels were increased in hippocampus of mice subjected to prolonged exercise [27]. βOHB accumulation in hippocampal neurons reduced the recruitment of HDAC2 and HDAC3 on brain derived neurotrophic factor gene (*Bdnf*) promoter, resulting in *Bdnf* increased transcription. BDNF is a well-known neurotrophic factor that improves learning and memory, thus βOHB could be the missing link that explains how exercise enhances mental abilities by counteracting anxiety and depression. Recently, it has been shown that βOHB induces β-hydroxybutyrylation of lysine residues (KBHB) in histones of cells exposed to high levels of βOHB. Moreover, histone KBHB levels increase in mouse livers during starvation or streptozotocin-induced diabetic ketosis, two conditions with elevated levels of βOHB. In these mice, the increased KBHB of histone H3 lysine 9 (H3K9BHB) is directly connected with upregulation of genes involved in starvation-responsive metabolic pathways. Therefore, this epigenetic modification is considered an important mechanism for adaptation to changes in cellular energy levels, impacting on epigenome modifications and modulating gene expression [28].

Taken together, all these evidences indicate βOHB as a ketone body with signaling functions that links outside environment to gene expression and cellular function. Consequently, its actions may have important implications for the pathogenesis and treatment of a variety of human diseases.

## 4. Clinical Relevance of Ketogenic Diet

The KD is the first line treatment for two metabolic disorders: glucose transporter 1 deficiency syndrome (GLUT1 DS) and pyruvate dehydrogenase deficiency syndrome (PDH DS). For these two metabolic disorders ketone bodies produced by KD represent an alternative energy source to bypass the defects related to these conditions. GLUT1 DS is an epileptic encephalopathy derived from the impairment of glucose transport across the blood–brain barrier due to GLUT1 dysfunction. GLUT1 is a membrane-bound glycoprotein with an established role mediating glucose availability in the brain [29]. Consequently, GLUT1 deficiency is associated to low glucose concentration in the cerebrospinal fluid and results in seizure, movement disorders and cognitive dysfunctions. So far, a variety of heterozygous mutations in GLUT1 gene have been described including large deletions, insertions, miss-sense and non-sense mutations [30], transmitted as a dominant autosomal trait [31]. However, no significant correlation between different mutations and clinical features was found [32]. In spite of the disease complexity, KD rapidly controls seizure episodes [33]. Furthermore, KD treatment provided in early infancy has been reported to prevent irreversible neuronal damage. In fact, GLUT1 DS is usually diagnosed in young children due to early clinical manifestations associated with the genetic defect and the KD should be started as soon as possible [34].

**Figure 3:**
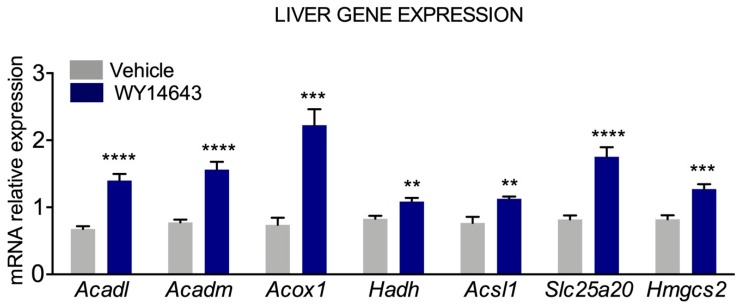
Treatment with peroxisome proliferator activated receptor α (PPARα) agonist, Wy14,643, induces ketogenic gene expression in *Glut1*^+/−^ mice. Hepatic expression of genes relevant for fatty acid β-oxidation (*Acadl, Acadm, Acox1, Hadh, Acsl1, Slc25a20*) and ketogenesis (*Hmgcs2*) in vehicle (grey bars) and Wy14,643-treated (blue bars) *Glut1*^+/−^ mice; ** *p* < 0.01; *** *p* < 0.001; **** *p* < 0.0001.

As previously mentioned, since PPARα plays an important role in the regulation of hepatic oxidative metabolism and ketogenesis, we hypothesized PPARα activation could increase ketone bodies availability in *Glut1*^+/−^ heterozygous mice, a well-established murine model of GLUT1 DS [35]. *Glut1* haploinsufficiency in mice, in fact, caused impaired motor activity, low levels of glucose in cerebrospinal fluid and electrographic seizure, thus resembling the main features of the human pathology. At this regard, we conducted a preliminary experiment with *Glut1*^+/−^ mice, kindly donated by Prof. De Vivo. We observed that treatment with Wy14,643, a PPARα ligand, increased hepatic expression of genes relevant for fatty acid β-oxidation, i.e., *Acadl, Acadm, Acox1, Hadh, Acsl1, Slc25a20* and for ketogenesis (*Hmgcs2*) (Figure 3). On the one hand, Wy14,643 treatment did not ameliorate motor performances on the rotarod test (preliminary data not shown). Still, latency on Rotarod at lower speed was not further worsened after Wy14,643 treatment, as opposed to vehicle treated mice, suggesting that activation of PPARα transcriptional network may prevent further neurological damage in *Glut1*^+/−^ mice. However, it should be noted that these observations are still preliminary and warrant further experiments to be confirmed. In addition, one limitation of our preliminary study is that the drug was administered to adult *Glut1*^+/−^ mice (16 weeks of age) when they have already developed neurological damage resulting in motor disorders. We cannot exclude that administration of drugs targeting PPARα at earlier stages of the disease could prevent the appearance of motor disturbances. If confirmed, these results could open possible avenues for the treatment of GLUT1 DS patients to prevent neurological damages and possibly allowing a more flexible diet regimen.

In PDH DS, pyruvate conversion to acetyl-CoA is compromised, resulting in infantile lactic acidosis, seizure and neurodegeneration [36]. Pyruvate dehydrogenase is a multi-enzyme complex mediating pyruvate oxidative decarboxylation to acetyl-CoA. A large number of mutations has been identified on pyruvate dehydrogenase gene and PDH DS results from mutations of E1 subunit for most of the patients [37,38]. PDH DS can also be secondary to other defects such as deficiency of lipoid acid, an essential PDH cofactor [39], or depletion of mitochondrial Lon protease 1, involved in mitochondrial metabolism reprogramming upon stress signals [40]. Acetate administration has been shown to decrease the duration of seizure episodes in PDH deficient mice, suggesting a link between brain metabolism and the regulation of neuron excitability [41]. In addition, KD successfully improves seizures and neurologic outcomes of patients [42]. Moreover, the dietary treatment is more effective on neuronal degeneration if started as early as possible after diagnosis. Epilepsy is one of the most common neurological disorder [43,44] and frequent seizure episodes are one of its most relevant clinical features. KD and its variants have been reported to effectively improve seizure frequency and severity in adults and children with drug-resistance epilepsy [45,46]. However, anti-convulsant effects are usually not achieved without continuing at least one anti-seizure drug. Thus, KD acts together with anti-epileptic drugs rather than alone to achieve proper seizure control in epileptic patients [47].

## 5. Biochemistry of KD Action

In spite of its clinical relevance in the treatment of epilepsy and of a number of other neurological and non-neurological diseases [48], the mechanism of KD action is not deeply understood. Therefore, significant efforts have been made to gain insights into the biochemistry of KD and different mechanisms have been proposed acting together by targeting different pathways. 

### 5.1. Inhibition of Glycolysis

KD induces a shift from carbohydrates to lipids as primary energy source, diverting body metabolism towards oxidation of ketone bodies instead of glycolysis. KD provides 90% of energy from fat and only 10% from proteins and carbohydrates combined [49]. Thus, the glycolytic pathway is limited due to carbohydrate restriction. In contrast, seizure is characterized by high glycolytic rate to provide energy necessary for seizure onset [50]. Therefore, inhibition of glycolysis is proposed as a mechanism contributing to the anti-seizure effect of KD. Moreover, the glycolytic inhibitor 2-deoxy-D-glucose (2DG) has been shown to exert acute and chronic anti-convulsant effects in different animal models of seizure and epilepsy [51]. When administered, 2DG competes with glucose as substrate for glycolysis in cells with a high energy demand. 2DG also blocks the glycolytic pathway since 2DG-6P cannot be further converted by phospho-glucose isomerase, resulting in inhibition of glycolysis [52]. It has been shown that the addition of 2DG reversibly reduces burst frequency after in vitro excitatory stimulation of hippocampal CA3 neurons [53]. Moreover, 2DG does not alter synaptic current amplitude suggesting a presynaptic role such as the regulation of vesicle release. Furthermore, the chronic anti-seizure effect of 2DG might be related to decreased NADH levels. Reduced NADH leads to transcriptional repression of factors involved in neuronal degeneration such as *Bdnf* and its receptor tropomyosin receptor kinase B (*TrkB*), as shown in the rat kindling model of epilepsy. BDNF and TrkB repression is mediated by the transcriptional repressor NRSF (Neuron Restrictive Silencing Factor) and its NADH sensitive co-repressor CtBP (Carboxy terminal Binding Protein), which do not dissociate from the promoter regions as a result of decreased NADH and maintains chromatin condensation at these sites [54].

### 5.2. Mitochondrial Metabolism

The energy for neuronal transmission is supplied by mitochondrial production of ATP through the electron transport chain [55]. Increased ATP levels within cells inhibit the neuronal ATP-sensitive potassium channels resulting in decreased neuron excitability. ATP-sensitive potassium channels and BAD (Bcl2-associated agonist of cell death) have been proposed as a molecular bridge between KD and anti-seizure effects [56]. BAD is known for its pro-apoptotic role through mitochondrial outer membrane permeabilization and cytochrome C release with subsequent activation of caspases and apoptosis [57]. Nevertheless, when phosphorylated on its BH3 domain, BAD also promotes mitochondrial oxidation of glucose [58,59] and, at the same time, inhibits ketone bodies utilization in neurons. Deletion or mutation of BAD in mice prevents its phosphorylation, shifts metabolism towards KB utilization and promotes seizure resistance. Moreover, mice with a deletion one subunit of the ATP-sensitive potassium channel in the *Bad* null background lose seizure resistance [56]. In addition, KD has been reported to reduce the mitochondrial production of reactive oxygen species (ROS) [60]. ROS production is physiological and it is controlled by endogenous antioxidant systems such as reduced glutathione (GSH). However, when production overcomes detoxifying systems, ROS accumulate resulting in oxidative stress, which is implicated in epilepsy genesis and progression [61]. KD has been reported to increase hippocampal GSH biosynthesis in rats, enhancing mitochondrial antioxidant capacity [62]. The basis of this action has been associated to NF E2-related factor 2 (Nrf2), a transcription factor activated by oxidative stress that upregulates genes involved in antioxidant pathways including the ones related to GSH synthesis and conjugation. In fact, after 3 weeks of diet, rats fed KD showed Nrf2 nuclear accumulation and increased activity of its target NAD(P)H: quinone oxidoreductase (NQO1) in the hippocampus [63]. Finally, mitochondrial biogenesis in the hippocampus has also been presented as a molecular mechanism of KD. Chronic ketosis activates a transcriptional program supporting de novo synthesis of mitochondria and results in enhanced ATP production [64]. ATP levels influence neuron membrane stability since ATP regulates transporters mediating membrane potential and homeostasis. This improves the maintenance of synaptic transmission and seizure resistance.

### 5.3. Modulation of Neuronal Transmission

Adenosine has been proposed as a molecular mediator contributing to the KD effects through regulation of neuronal excitability. In particular, increased ATP levels due to KD raise adenosine production. Elevated adenosine in the synapse, in turn, enhances the activation of its inhibitory receptor A1R, reducing neuron excitability and seizure. In fact, both A1R knockout mice and genetically modified mice presenting adenosine kinase gain of function develop seizure episodes [65]. Furthermore, diet-derived ketone bodies have been proposed to modulate glutamate and GABA metabolism, promoting GABA inhibitory neurotransmission and seizure resistance. First, ACA has been shown to directly modulate GABAa receptors. In fact, ACA administration in rabbits after exposure to thujone, a GABAa receptor antagonist, produced anti-convulsant effects [66]. In addition, ACA and βOHB have been reported to increase GABA concentration within rat brain synapsis [67]. In particular, acetyl-CoA derived from ketone bodies condensates with oxaloacetate for incorporation into TCA cycle. Consequently, glutamate transamination into aspartate is reduced and more glutamate is available as substrate for glutamic acid decarboxylase (GAD), the enzyme mediating GABA biosynthesis [68]. In fact, in vivo administration of ACA or βOHB increased glutamate synaptic levels, reduced aspartate concentration and raised GABA biosynthesis in rat brain [67]. Moreover, βOHB and ACA have been shown to directly compete with Cl^-^ for allosteric activation of glutamate transporters, reducing glutamate release and excitatory synaptic transmission in rat hippocampus [69]. However, in vitro studies did not confirm ACA and βOHB effects on glutamate and GABA transmission in cultured hippocampal neurons [70]. Moreover, caloric restriction independent of ketogenic effects has been demonstrated to increase the expression of *Gad* brain main isoforms and GABA synthesis in rats [71]. Finally, ketosis did not alter glutamate and GABA levels in mice fed with KD [72]. Thus, whether ketone bodies modulate GABA and glutamate transmission remains controversial and needs further investigation.

### 5.4. Regulation of Polyunsaturated Fatty Acid Levels

KD has been shown to increase serum levels of polyunsaturated fatty acids (PUFAs) such as linoleic acid and docosahexaenoic acid (DHA). Such changes of PUFA levels correlated with seizure control [73]. In addition, n-3 PUFAs have been reported to exert anticonvulsant effects in rats after pharmacological seizure induction [74]. Since PUFAs are known to activate PPARs, the molecular basis of their action has been associated to these nuclear receptors that regulate transcription of genes involved in several biological processes including energy metabolism and neuronal excitability. In particular, PPARα activation has been shown to be a relevant molecular pathway. Lithium pilocarpine-induced epileptic rats fed with KD containing fenofibrate, a PPARα agonist, did not show additional anticonvulsant effects compared to the groups fed only with KD or fenofibrate, suggesting that both KD and fenofibrate activate the PPARα-mediated transcriptional pathway [75]. PPARγ has also been reported to be activated by KD in a mouse model of seizure [76]. In particular, PPARγ activation suppressed the expression of cyclooxygenase *Cox2* and microsomal prostaglandin E2 synthase-1 (*mPGES-1*) in the hippocampus. Moreover, hippocampal TNF-α was reduced. Thus, PUFAs might contribute to seizure resistance through the resolution of inflammatory processes in the brain. It should be noted that PUFAs are precursors of anti-inflammatory lipid mediators [77,78]. Nonetheless, PUFA role in KD action is debated since clinical trials have shown controversial outcomes. PUFA oral supplementation for six months improved frequency and strength of seizures in spite of the small number of patients included in this trial [79]. Furthermore, 12-weeks of oral supplementation with eicosapentaenoic acid (EPA) and DHA resulted in reduced seizure frequency of epileptic patients for the first weeks, but no amelioration was observed after 6 weeks, despite increases in EPA and DHA serum levels [80]. Furthermore, 12 weeks of PUFA supplementation did not show significant differences compared to placebo in terms of patient seizure frequency [81]. Thus, further investigations will be necessary to define PUFA role in KD action and seizure control and the dose PUFA administered to patients might need to be refined [82].

## 6. Ketone Bodies in Inflammation and Immune System

Ketone bodies are now considered modulators of inflammation and innate immune response. In particular, βOHB regulates the activation of the NLRP3 inflammasome in neutrophils and macrophages and blocks the activation of this specific inflammasome in response to pathogen associated molecular patterns (PAMPs) and damage associated molecular patterns (DAMPs) [83]. Interestingly, the suppressive effect is not related to Gpr109a, a GPCR-mediating βOHB signaling, nor to the inhibition of HDACs. In lipopolysaccharide (LPS)-primed mouse bone marrow-derived macrophages (BMDMs) incubated with NLRP3 activators (i.e., ATP, monosodium urate (MSU) and ceramide), βOHB blocked the K^+^ efflux from the cytosol, which triggers the activation of NLRP3 inflammasome and of the downstream assembly of the inflammasome complex. Consequently, reduction in caspase-1 activation and secretion of pro-inflammatory cytokines IL-1β and IL-18 was observed. These results were confirmed by experiments in human monocytes and mouse models of NLRP3-related autoinflammatory syndromes, i.e., Muckle–Wells syndrome (MWS) and familial cold autoinflammatory syndrome (FCAS). Mouse models with constitutively active human NLRP3 were fed KD and showed increased βOHB serum levels and protection from neutrophilia typical of these disease states, although they did not show effects on frequency and recruitment of immune cells. Thus, authors suggest that in patients affected by autoinflammatory syndromes, KD could be adopted in order to ameliorate the therapeutic outcome [83]. Consistent with these findings, KD and related increase in βOHB may be useful in the treatment for gout, an inflammatory arthritis caused by MSU crystal accumulation in joints. KD showed efficacy in gout mouse model of all ages and did not decrease immune host response to infections [84].

Anti-inflammatory effects of KB could also decrease cardiovascular risk by reducing low-grade chronic inflammation that characterize cardiovascular (CV) disease. Administration of oral anti-hyperglycemic medications sodium/glucose cotransporter (SGLT) 2 inhibitors to type 2 diabetic patients showed greater efficacy vs. other glucose-lowering drugs. Glycated haemoglobin (HbA1c) levels were reduced with both classes of drugs, but SGLT 2 inhibitors exhibit a more pronounced cardioprotective effect. This could be accounted to the elevation of βOHB plasma levels up to mM concentrations in diabetic patients treated with SGLT 2 inhibitors, as hypothesized by the authors [85].

βOHB exhibited an anti-inflammatory effect also in renal ischemia/reperfusion injury (IRI). This effect occurs by regulating pyroptosis, a type of caspase-1 dependent programmed cell death distinct from apoptosis and necrosis. Block of pyroptosis is dependent on the upregulation of FOXO3 expression mediated by βOHB, as previously discussed [24]. While high concentrations of βOHB cause ketoacidosis, a risk factor for developing acute kidney injury (AKI), concentrations in the range of mM seemed to promote tissue protection in IR-injured mice by decreasing Terminal deoxynucleotidyl transferase dUTP nick end labeling (TUNEL)-positive kidney cells [86].

Comprehensively, these results provide preclinical evidences for possible adoption of KD as adjuvant in the treatment of several diseases associated with inflammation. However, clinical trials are needed to ascertain the effectiveness of this strategy in patients.

## 7. Ketogenic Diets as Anti-Cancer Adjuvant Therapy to Target Metabolic Rewiring

**Figure 4:**
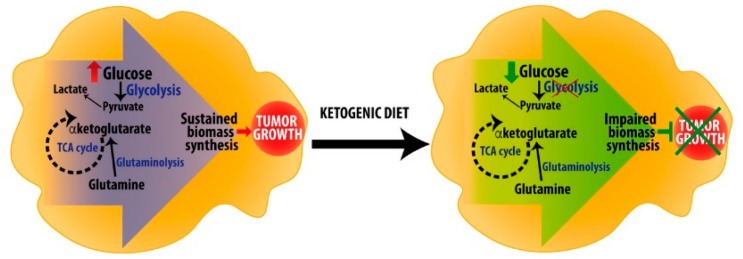
Hypothetical role of ketogenic diet in cancer metabolic rewiring. Cancer cells rely upon metabolic rewiring characterized by increased glucose uptake (red arrow) and glycolysis, increased glutaminolysis and TCA cycle rates, which sustain biomass expansion and tumor growth. Ketogenic diet, by reducing glucose oxidation (green arrow), does not further support cancer cells proliferation, thereby impairing tumor expansion.

Considering the pleiotropic effects of ketone bodies, from metabolism to signaling, ketogenic diet is now considered a possible therapeutic approach in many diseases, besides epilepsy. Metabolic reprogramming in cancer cells is intensely studied to detect new possible targets to starve cancer cells leading to tumor growth arrest. Because of Warburg effect, tumor cells rely on glucose as a primary source for energy and for biomass production. On the other hand, ketogenic diets, by reducing glucose concentration in blood and providing ketone bodies to normal tissues, may lead to metabolic reprogramming and affect cancer cells survival and proliferation, especially in highly glycolytic tumors (Figure 4) [87].

KD has been considered a possible therapeutic approach for malignant glioma and other brain tumors, characterized by metabolic reprogramming and with unfavorable prognosis [88]. A systematic review of the effects of ketone supplementation, ketogenic diet, calorie restriction and short-term starvation in treatment of malignant gliomas found a trend toward a correlation between reduced blood glucose and reduced tumor growth and improved survival in preclinical studies [89]. Synergistic effects were also observed when these diets were used in combination with conventional therapies. However, clinical data are still insufficient to draw clear conclusions on efficacy of ketogenic diets. De Feyter et al. [90] reported that the degree of ketone oxidation of glioma cell lines, implanted in rats, did not differ from surrounding non-tumor tissue, and that KD had no effects on tumor growth and survival, suggesting that cancer cells are actually able to use ketones to support their growth. Furthermore, authors reported that expression of *Mct1*, the monocarboxylate transporter for ketone bodies, increased in the blood–brain barrier following KD, indicating that metabolic rewiring may lead cancer cells to grow under glucose starvation conditions. Consequently, the evaluation of ketolytic enzymes expression in tumor biopsies may not predict which tumors may favorably respond to KD as metabolic adaptation may occur only after exposing tumors to ketogenic conditions. Interestingly, the expression of glucose transporter isoform *GLUT3* strongly correlates with the outcome of brain tumor patients [91]. In particular, *GLUT3* expression correlated with tumor grade and its expression was increased in tumor recurrence and in specimens of patients died within three years. *GLUT3* was the only isoform found to negatively impact prognosis on survival at one year and in the long term (more than 3 years). The same authors showed that brain initiating tumor cells (BTIC), which are thought to be responsible of sustained tumor growth and tumor recurrence, are promoted in glucose deprivation condition. Moreover, upon glucose disposal non-BTIC cells acquire BTIC phenotype, suggesting that tumor may adapt to glucose deprivation. Indeed, another study demonstrated that KD treatment after tumor initiation was not able to reduce hepatic tumor incidence, tumor multiplicity and tumor volume in DEN (diethylnitrosamine)-treated mice, a common model of liver tumorigenesis [92]. Accordingly, a meta-analysis of studies using murine models of cancer showed that ketogenic diet exerted a positive effect on mean survival [93], with initiation of dietary treatment and/or tumor location being the parameters influencing KD efficacy on survival. Interestingly, this study also highlighted that KD may be used as an approach for secondary prevention, i.e., to reduce the risk of tumor recurrence. Therefore, taking into account the lack of clear-cut evidence of KD efficacy against tumor growth, KD is considered more useful as a support dietary treatment in adjunction to classical anti-cancer therapies [94]. Klement [95] reported that in 49% of human studies there were evidences of KD anti-tumor effects, with only one study reporting evidences of pro-tumorigenesis after KD treatment. Moreover, 50% of these studies reported quality of life amelioration in KD treated cancer patients in terms of improvement of general conditions and control of neurological function and seizure in patients bearing brain tumors. This review emphasized that, despite KD treatment was not always effective in reducing blood glucose levels, the rise in ketone bodies and free fatty acids itself has an inhibitory effect on glycolysis. However, from this precise analysis emerged that clear evidences for KD anti-tumor effects are lacking in the framework of human pathology. Nevertheless, Klement stated that “KD can be safely offered to cancer patients” as the probability of gaining beneficial effects is still higher than the opposite. Importantly, in a very recent randomized controlled trial endometrial and ovarian cancer patients were subjected to KD for 12 weeks which resulted in reduced fat mass with no change in lean mass and reduced levels of fasting insulin and C-peptide, factors involved in sustaining tumor growth especially during obesity [96]. Moreover, KD treatment also improved physical function and decrease cravings of specific food, which may help to improve quality of life [97]. Ok et al. [98] showed that KD improved meal compliance and increased energy intake rates in pancreato-biliary cancer patients after surgery, which are crucial parameters for life quality and survival, especially for patients undergoing chemotherapy after surgery, therefore this study suggests KD as a safe adjuvant therapy for pancreato-biliary cancer patients. However, it is also important to remember that side effects emerging from the use of KD in combination with anti-cancer drugs has not been fully elucidated yet [87]. In conclusion, the efficacy of ketogenic diet in reducing cancer growth is not fully established yet. Perhaps the ketogenic diet may be considered to be applicable to cancer therapies to improve patients’ quality of life, helping them to cope the burden of chemo- and radiotherapies.

## 8. Could Ketogenic Diet Help in Treating Neurodegenerative Diseases?

As discussed previously, KB exert neuroprotective effects via promotion of mitochondrial activity and reduction of oxidative stress. KB also exhibit anti-apoptotic properties and may help in stabilizing the functions of the synapses [99]. Therefore, KD has been considered as a possible treatment for neurodegenerative diseases, like Alzheimer’s (AD) and Parkinson’s (PD) diseases. In a clinical trial, Taylor et al. [100] demonstrated that 3 months KD is a feasible and safe intervention in AD patients, especially those with very mild or mild dementia. Despite small sample size, authors observed an improvement in cognitive scores after dietary intervention, which returned at the baseline after washout. However, in this trial all enrolled AD patients with moderate dementia withdrew the study and their poor compliance to the dietary intervention was ascribable to increased caregiver burden, suggesting that these interventions in moderate to severe dementia patients should probably require implementations of specific medical care actions. Moreover, other approaches increasing KB synthesis are currently investigated. For instance, authors showed results from long-term treatment of AD patients with caprylidene [101]. This molecule is the triacylglycerol form of caprylic acid that, once metabolized, produces βOHB and ACA. Long-term treatment with caprylidene increases cerebral blood flow in the left superior lateral temporal cortex in patients negative for ApoE-ε4 risk allele, suggesting increased brain metabolism in this area. In another trial, AC-1202, a type of medium chain triacylglycerol, which increases KB levels, was applied to a larger number of AD patients for 90 days without other dietary interventions [102]. AC-1202 induced mild ketosis and improved cognitive parameters (changes from baseline in ADAS-Cog score) only in AD patients not bearing the risk allele ApoE-ε4. However, Broom et al. [103] highlighted that reduction of carbohydrates should be considered important not only because it may improve ketone bodies synthesis but also because it prevents glycation of proteins, such as ApoE, responsible for lipid trafficking in the brain.

In the context of PD, an early study in 2005 showed that five patients following KD for 28 days improved their UPDRS score (Unified Parkinson’s Disease Rating Scale), an international system that scores both motor and non-motor symptoms [104]. However, as also stated by authors, a placebo effect should not be excluded, possibly due to the limited number of patients enrolled in the study. Recently, Phillips et al. [105] compared low-fat and KD interventions in PD. They demonstrated that both interventions are feasible and safe and both diets improved MDS-UPDRS score. Interestingly, KD-treated patients showed a greater improvement of non-motor symptoms, which are less responsive to L-DOPA treatment and probably the most disabling symptoms of PD. Of note, the most important adverse effect reported by the KD-treated group was a transient exacerbation of tremor and/or rigidity that mostly resolved by the end of the trial. Therefore, this study suggested that KD can be applied as a feasible and effective treatment in conjunction with normal therapy in PD patients. Besides, KD has proven to exert positive effects in other neurodegenerative diseases with alteration of motor function, at least in preclinical models, as reviewed elsewhere [106]. Interestingly, it has been demonstrated that KD increases longevity and health span in adult mice [107]. In fact, 12 months aged mice were fed either control, low carbohydrate or KD and only KD feeding resulted in increased lifespan, increased cognitive and motor performances and reduced incidence of tumor. Of note, mice were fed in isocaloric amounts. In another work, mice were fed *ad libitum* a cycling KD regimen: one week KD feeding was alternated to one week control diet feeding [108]. The cycling KD regimen positively affected healthspan and improved memory in aging mice. However, in this case, authors only detected a protective effect in midlife mortality, not overall survival. Moreover, both cycling KD and cycling low-carbohydrate high fat non-ketogenic diet showed decreased glucose metabolism, fatty acid and protein synthesis in the liver. However, only cycling KD showed a strong upregulation of PPARα regulatory networks. Considering the increasing burden of aging-related diseases, these studies suggest that KD and its related metabolic rewiring may be a suitable target of investigation in order to prevent aging-related impairments and to promote healthspan.

## 9. Ketogenic Diet as a Strategy to Cope Obesity

Another interesting area of research in this field is the application of ketogenic regimen as treatment for obesity and related metabolic dysfunctions. Since the prevalence of obesity is increasing, many efforts are currently spent to find strategies to cope with this condition that seriously impacts health and quality of life. Interestingly, in a meta-analysis review Mansoor et al. [109] showed that consumption of low carbohydrate diets in overweighed “healthy” subjects induced a greater weight loss when compared with individuals following low fat diets. However, low carbohydrate diets also increased levels of LDL-cholesterol, which is a well-known cardiovascular risk factor, therefore it was not possible to draw clear conclusions for potential advantages of low carbohydrate diets vs. low fat diets on cardiovascular risk factors. The meta-analysis from Bezerra Bueno et al. [110] on long term very low carbohydrate ketogenic diet (VLCKD) interventions for obese patients showed similar results. However, authors underlined that adherence to dietary interventions was low and that differences, even though statistically significant, could be “of little clinical significance” in the long term when compared to conventional therapies. Authors suggested to carefully consider pro and cons of VLCKD before recommending it as treatment for obesity. More evidences are, therefore, required to demonstrate whether KD is effective to cope obesity and related cardiometabolic dysfunctions. Very recently, it has been shown that non ketogenic low and medium low carbohydrates diets are as effective as KD in reducing body weight, BMI, waist and hip circumference in healthy subjects [111]. Interestingly, these dietary interventions reduced insulin blood concentration. However, also in this study adoption of low carbohydrate high fat diets increased total cholesterol and LDL-cholesterol, which were counterbalanced by increased HDL-cholesterol and reduced triglycerides. Therefore, carbohydrate restriction may improve cardiometabolic parameters in healthy subjects and it is easily achievable compared to very low carbohydrate ketogenic diets. Nonetheless, the increase in LDL-cholesterol emphasizes that these dietary interventions may not be suitable for all subjects and that careful considerations on health status and lipidaemia should be recommended before adopting such diets. Interestingly, another metanalysis calculated atherosclerotic cardiovascular disease risk prediction and reported that both low carbohydrate and low fat diet were associated with weight loss and reduced cardiovascular risk with low carbohydrate diet favoring a mild, though statistically meaningful, greater extent of risk reduction [112]. This metanalysis also highlights that, although current guidelines suggest low fat diets as the preferential diet intervention to reduce overweight and cardiovascular risk factor, policy makers should take in consideration that low carbohydrate diets may have a beneficial role in the management of obesity. From the results presented herein, it appears that different dietary approaches are currently available to cope obesity. However, advantages of one approach over the others are not clear. Furthermore, it appears that the full consideration of patient individual metabolic condition could help to decide the best dietary intervention that may result in metabolic improvement.

KB metabolism has been evoked in the pathogenesis of one of the metabolic disturbances frequently associated with obesity: non-alcoholic fatty liver disease (NAFLD). This disease is characterized by the accumulation of extra fat in the hepatocytes and it may evolve to steatohepatitis (NASH). Ketogenesis is impaired during NAFLD progression in animal models [113]. The same authors demonstrated that 8 weeks of HFD feeding in *Hmgcs2* knockdown mice induced liver injury, increased macrophages and stellate cell activation, which resemble features of NASH, thus suggesting impaired ketogenesis as typical metabolic trait of progression to NASH [114]. Ketogenesis insufficiency led to increased deviation of acetyl-CoA to de novo lipogenesis via citrate shuttling. Moreover, TCA cycle and gluconeogenesis are impaired in HFD state as a result of reduced CoASH availability. In a follow-up study, it has been demonstrated that during HFD feeding ketogenesis, as long as hepatic glucose production, is increased in the liver, but this pathway was not capable to sufficiently dispose lipid load [1]. When ketogenesis was genetically inactivated (*Hmgcs2* knock down mice), hepatocytes became primed to store glycogen in the fed state, as it appears from reduced levels of total and phosphorylated form of glycogen phosphorylase. Ketogenic insufficient mice also displayed increased TCA cycle, not counterbalanced by anaplerosis, increased gluconeogenesis and pyruvate cycling and accumulation in hepatocytes of bis(mono-acyl-glycero) phosphates, lipid species associated with late endosome. With respect to human pathology, Browning et al. [115] showed that 2 weeks calorie and carbohydrate restriction intervention in NAFLD patients was more effective in reducing hepatic triglyceride content than calorie restriction alone. Authors also suggested that this effect was ascribable to increased hepatic and whole-body fat oxidation in response to carbohydrate restriction. These reports consistently highlight that ketogenesis impairments may lead to progression of NAFLD into NASH. From these recent evidences, it appears that this pathway may be a suitable target to prevent NASH.

## 10. Adverse Effects of Ketogenic Diet

KD is considered a safe strategy for the treatment of a number of diseases. Nevertheless, a profile of adverse events has been described in relation to KD. Mild gastrointestinal dysfunctions are the side effect reported for most of the patients under KD [116]. In addition, dyslipidemia might occur during KD administration [117]. In particular, serum cholesterol and triglycerides increased during the diet in a prospective study including adults with refractory epilepsy [118]. In addition, several data available from children and those of a younger age are related to major risk of side effects [119]. A decline in linear growth with the KD has been described in patients on the diet for more than six months; the percentage of affected patients varies between 6% and 30% in recent long term studies [120,121,122,123]. Moreover, differences in skeleton development have been observed in relation to vitamin D deprivation and the possible association with increased fracture risk in adulthood needs to be monitored [124]. Furthermore, kidney stones are associated with KD in several pediatric patients [125]. Finally, malnutrition and weight loss are not easily tolerated also by older patients affected by neurodegenerative diseases such as AD or PD [99].

## 11. Conclusions

βOHB, the most abundant ketone body, is itself not only an energetic metabolite but also a signaling molecule that integrates the metabolic status of the cell with epigenetic regulation of nuclear function as well as a regulator of the inflammatory response. Many studies published in recent years shed light on the tight interconnection between metabolism and the function of the cell and its organelles. Therefore, disturbances in metabolic pathways may be virtually involved in the pathogenesis of any disease. For instance, this concept is demonstrated by the increasing amount of data underlying the importance of metabolic rewiring in the onset and development of cancer and neurological disorders. Considering that the effects of KD relies on the tight regulation of two opposing pathways (ketogenesis and ketolysis), a deeper understanding of the biochemical basis of their regulation is needed to fine-tune the use of this dietary treatment and unravel its long-term effects. The regulation of ketogenesis and ketolysis has been integrated by new data, shedding light on novel aspects such as circadian rhythms, food intake behavior, and paracrine signals of regulation. The combination of classical methodologies with new technologies (e.g., -omics and bioinformatics, in vivo fluxomics by nuclear magnetic resonance, NMR) allowed new aspects of the regulation of KB metabolism to unravel. As a fasting-mimicking diet, KD is currently being considered for application not only to epilepsy but also to cancer, neurological diseases, and metabolic disorders like T2D, obesity, and CV disease. In these diseases, inflammation is a common hallmark and KB have been shown to display anti-inflammatory properties [83]. In the context of cancer, there is evidence that tumor cells may rewire metabolism in order to survive and grow in the presence of limited energy sources. Therefore, it is not totally clear whether the use of KD in combination with conventional therapies may favor or not prognosis. Based on the available data, KD may have a potential role as adjuvant therapy to limit side effects of chemotherapy and to reduce pro-tumorigenic factors. Evidence for KD efficacy in neurological disorders is also limited. Preclinical investigations in animal models for these diseases may help to unravel the pathogenetic role of metabolic alterations and how metabolic rewiring induced by KD may slow down the progression of neurological disorders. We also considered the possible adoption of KD in obesity. However, the potential of KD as a new strategy to cope with obesity should be further investigated before suggesting it in dietary recommendations.

A key issue is the management of the KD regimen in the everyday life of patients. The limited choice of ingredients, due to the high content of lipids and low amount of carbohydrates and proteins, represents a hurdle to reach adequate compliance of patients and makes KD difficult to manage. Therefore, it would be necessary to provide caregivers with more resources to ensure adherence to this diet. Finally, long-term metabolic consequences of the adoption of a diet enriched in fat remain to be fully elucidated. Carefully designed clinical studies with larger patient populations would help clarifying whether KD could be successfully applied to disorders with a metabolic basis and to address the issue of long-term consequences of this diet regimen.
